# Rapid Modulation of Distributed Brain Activity by Transcranial Magnetic Stimulation of Human Motor Cortex

**DOI:** 10.1155/2006/287276

**Published:** 2006-11-27

**Authors:** Lucy Lee, Hartwig Siebner, Sven Bestmann

**Affiliations:** ^1^Wellcome Department of Imaging Neuroscience12 Queen SquareLondonWC1N 3BGUK; ^2^Department of NeurologyChristian-Albrechts-UniversityKielGermany; ^3^NeuroImageNord Kiel-Hamburg-Lübeck at Hamburg University HospitalHamburgGermany

## Abstract

This paper reviews the effects of single and repetitive transcranial magnetic stimuli (rTMS) delivered to one cortical area and measured across distributed brain regions using electrophysiological measures (e.g. motor thresholds, motor evoked potentials, paired-pulse stimulation), functional neuroimaging (including EEG, PET and fMRI) and behavioural measures. Discussion is restricted to changes in excitability in the primary motor cortex and behaviour during motor tasks following transcranial magnetic stimulation delivered to primary motor and premotor areas. Trains of rTMS have lasting effects on the excitability of intrinsic and corticofugal neurones, altering the responsiveness of local and remote sites. These effects lead to distributed changes in synaptic activity at rest, and during a range of motor tasks. It is possible to impair or improve performance following rTMS, but for most simple motor tasks performance is unaltered. Changes in distributed activity observed with functional imaging during motor behaviour may represent compensatory activity, enabling maintenance of performance; stimulation of additional cortical areas appears to impair performance. A detailed understanding of the distributed changes in excitability following rTMS may facilitate future attempts to modulate motor behaviour in the healthy brain and for therapeutic purposes.

## 1. Introduction

The aim of this paper is to provide an overview of the effects of transcranial magnetic stimuli (TMS) in areas remote from the site of stimulation. When using a standard figure-of-eight coil the strength of the magnetic field produced during a TMS pulse decreases progressively with distance from the centre of the coil; effective direct stimulation occurs in a limited area of cortex close to the centre of the coil [2,70]. Any effects of TMS seen outside this area are thought to be mediated by cortico-cortical and cortico-subcortical-cortical connections. This review forms part of a series of papers discussing the use of transcranial magnetic stimulation (TMS) to disrupt the brain with the aim of enhancing human cognitive abilities. The focus of this paper will be restricted to studies of TMS in the human motor system. Studies of the motor system offer a unique opportunity to combine data from different techniques in order to establish a more complete characterisation of the interaction between local and remote effects of TMS on excitability and behaviour.

The effects of TMS on the excitability of the motor system can be assessed directly by measuring motor evoked potentials (MEPs) or indirectly by functional neuroimaging (e.g. positron emission tomography (PET), functional magnetic resonance imaging (fMRI), electroencephalography (EEG)) and measures of motor behaviour (e.g. force generation, movement velocity, movement accuracy, reaction times, response accuracy, sequence learning). Each of these methods has strengths and weaknesses. Electrophysiological techniques provide a direct, objective measure of cortical and/or corticospinal excitability. The first sec-

ISSN 0953-4180/06/$17.00 © 2006 – IOS Press and the authors. All rights reserved tion introduces methods available for assessing the excitability of the motor system, and reviews studies using such techniques to demonstrate that TMS delivered to one area of the motor system alters the excitability of a remote area. These methods use the size of the motor evoked response to characterise changes in corticomotor excitability. However, it is only possible to obtain direct measures of excitability from primary motor cortex and to examine changes in the responsiveness of the primary motor cortex to inputs from distant motor areas. It is not possible to assess the excitability of sub-cortical structures, neither is it possible examine the effects of changes in the excitability of primary motor cortex on other areas. Functional neuroimaging enables the characterisation of TMS effects on synaptic activity throughout the brain, both at rest and during task performance. However, functional neuroimaging does not provide a direct measure of excitability. The role of functional neuroimaging in evaluating the distributed effects of focal TMS is discussed in section two. Perhaps most importantly in the context of improving brain function neither directly measured changes in cortical excitability nor changes in synaptic activity measured with functional neuroimaging necessarily translate into improvements or impairments of task performance. Behavioural correlates of motor function, such as changes in reaction times, performance accuracy, and rate of learning can provide this information. However, there are several potential problems with the interpretation of changes or the lack of changes in such outcome measures. These are discussed in section three.

The results of studies using a combination of the methods outlined above are discussed in section four. Studies of the motor system offer a unique opportunity to examine the effects of TMS because electrophysiological, metabolic and behavioural measures can readily be combined. Examining the relationships between distributed changes in activity caused by TMS and changes in task-related activity during periods of abnormal cortical/subcortical excitability reveal interesting features of the motor system. Specifically, the motor system appears to be able to adapt quickly to TMS induced changes in the excitability and effective connectivity of the areas engaged in a motor task, maintaining task performance. This may explain why attempts to impair or improve motor performance with TMS have had limited success, and to point to future directions of research that may be more successful. These issues are addressed in section five.

## 2. Measuring and modulating the excitability of the motor cortex and corticospinal tract with TMS

TMS can be used in a variety of ways to measure the excitability of the motor cortex and corticospinal tract. Several comprehensive reviews describe the different techniques and their biophysical and physiological mechanisms [11,20,86,94].

In brief, the motor threshold (MT) describes the lowest intensity of TMS that can elicit a motor evoked potential of approximately 50 $\mu$V. This is thought to reflect neuronal membrane excitability because it is increased by drugs that alter membrane conductance [14,93]. It can be measured in resting or activated muscles (RMT and AMT respectively). The RMT represents the intensity of stimulation required to activate cortico-spinal neurones, and therefore it is assumed that stimulation at or above the RMT will also activate cortico-cortical connections [71]. Given the focus of this review on distributed effects of TMS, we will use the RMT to define the threshold of stimulation delivered in the studies described below. Subthreshold stimulation refers to stimulation given below RMT, whereas suprathreshold describes stimuli at intensities at or above RMT i.e. eliciting muscle twitches.

The amplitude of the motor evoked potential (MEP) elicited from a peripheral muscle represents a measure of the excitability of the cortex, sub-cortex and spinal tract. The silent period (SP) describes a short cessation of muscle activity when a single pulse of TMS is delivered during voluntary muscle contraction. The duration of the late phase of the SP provides a measure of the excitability of cortical inhibitory interneurons (presumably GABAb-ergic) [15,28].

Further characterisation of cortical excitability can be obtained with paired-pulse TMS using a conditioning-test paradigm. The most frequently used paired-pulse paradigm [38] gives a subthreshold conditioning stimulus to condition the MEP amplitude elicited by a subsequent suprathreshold test stimulus. At inter-stimulus intervals (ISIs) of 1–5 ms the amplitude of the test MEP is inhibited (intracortical inhibition: ICI), and at longer ISIs (8–30 ms) the response is facilitated (intracortical facilitation: ICF). These phenomena appear to occur in the cortex [22,23,38,56]. GABA agonists and glutamatergic antagonists increase ICI and decrease ICF whereas drugs that alter membrane conductance have no effect [42,93,95].

### 2.1. Intra-hemispheric effects of TMS

The most rapid modulation of cortical excitability by TMS occurs within milliseconds of stimulation. Using a variation of the paired-pulse techniques described above Civardi et al. [18] demonstrated that at an ISI of 6 ms stimulation of the premotor (PM) cortex (3–5 cm anterior to the motor hand area) with sub- and suprathreshold conditioning stimuli lead to inhibition and facilitation of the amplitude of MEPs elicited from the ipsilateral cortex hand area, respectively.

Modulating the excitability of the premotor cortex with rTMS can induce more enduring changes in the excitability of the ipsilateral primary motor cortex (M1). 1 Hz rTMS to M1 and dorsal premotor cortex (PMd) reduces the amplitude of MEPs elicited from M1 [13, 30]. 5 Hz rTMS to M1 and PMd increases the amplitude of MEPs elicited from M1 [63,68]. The direction of distributed changes in cortical excitability appears to be frequency-dependent (see Fig. 1) and the direction of effects on MEP amplitude appears to be similar for M1 and PMd stimulation. However, other measures of corticocortical excitability such as paired-pulse excitability may not follow such a straightforward relationship. For example, subthreshold 1 Hz rTMS to PMd increases ICF at 7 ms for up to one hour [55] whereas subthreshold 5 Hz rTMS decreases ICF in M1 [68]. Conversely, subthreshold 1 Hz rTMS to M1 decreases ICF [69] whereas 5 Hz rTMS to M1 decreases ICI but does not affect ICF [62].

### 2.2. Inter-hemispheric effects of TMS

Stimulation of the primary motor cortex has immediate effects on the excitability on distributed brain areas, the most readily measured of which being the contralateral homologous motor cortex. Cracco et al. [19] first demonstrated TMS-evoked transcallosal responses and Ferbert et al. [24] subsequently showed that conditioning stimuli delivered to one motor cortex inhibited MEPs elicited from the contralateral motor cortex 5–6 ms later. Lower intensity conditioning stimuli to motor and ventral premotor cortices also inhibit contralateral test MEPs and somatosensory evoked potentials (SEPs)s [51]. Other studies report inhibitory interactions between homologous primary motor hand areas whereby silent periods are induced in tonically active muscles ipsilateral to the site of stimulation [48, 49]. Subtle facilitatory interactions have been reported between the primary motor hand areas at ISIs (4–5 ms) with inhibition occurring at longer ISIs [33,89]. For a more complete review of this area see Chen et al. [12].

rTMS to the primary motor cortex can alter the excitability of the contralateral motor cortex, and the magnitude of interhemispheric effects, for longer periods of time. Recent studies have shown opposing effects of 1 Hz rTMS on the excitability of the stimulated and contralateral hemispheres. Suprathreshold 1 Hz rTMS decreased MEP amplitude in the stimulated hemisphere while decreasing intracortical inhibition [64] and increasing the slope of MEP recruitment curves [73] in the contralateral hemisphere. Gilio et al. [31] found increased MEP amplitudes in the contralateral hemisphere and a reduction of the early phase of paired-pulse inhibition from the stimulated (conditioned) to the non-stimulated (test) hemisphere. Using a series of control experiments, these authors concluded that these effects were predominantly mediated by cortico-cortical circuits rather than spinal mechanisms or afferent feedback. A comparison of 0.5 Hz and 5 Hz subthreshold rTMS on the excitability of the contralateral hemisphere revealed increased MEP amplitude with 5 Hz rTMS, but no change with 0.5 Hz [32].

The exact mechanisms of interhemispheric effects of TMS are incompletely understood. Direct transcallosal projections between the motor representations of the hand are sparse [72] but rTMS to the primary motor cortex undoubtedly results in a range of lasting changes in the excitability of distributed brain areas that is mediated via direct corticocortical [21] or cortico-subcortical-cortico pathways [29].

## 3. Measuring the effects of TMS on synaptic activity

The techniques outlined in Section 1 only monitor excitability changes in the primary motor cortex or changes in the responsiveness of the primary motor cortex to inputs from distant motor areas. Functional neuroimaging enables the visualisation of TMS effects on synaptic activity throughout the brain, both at rest and during task performance. However, it does not provide a direct measure of excitability. Electroencephalography (EEG) measures mean postsynaptic potentials in the neocortex. EEG data can be analysed to provide information about local changes in postsynaptic potentials during a specific event (event related potentials: ERPs), amplitude changes in defined frequency bands (power spectra analysis) and changes in the phase consistency between pairs of signals in each

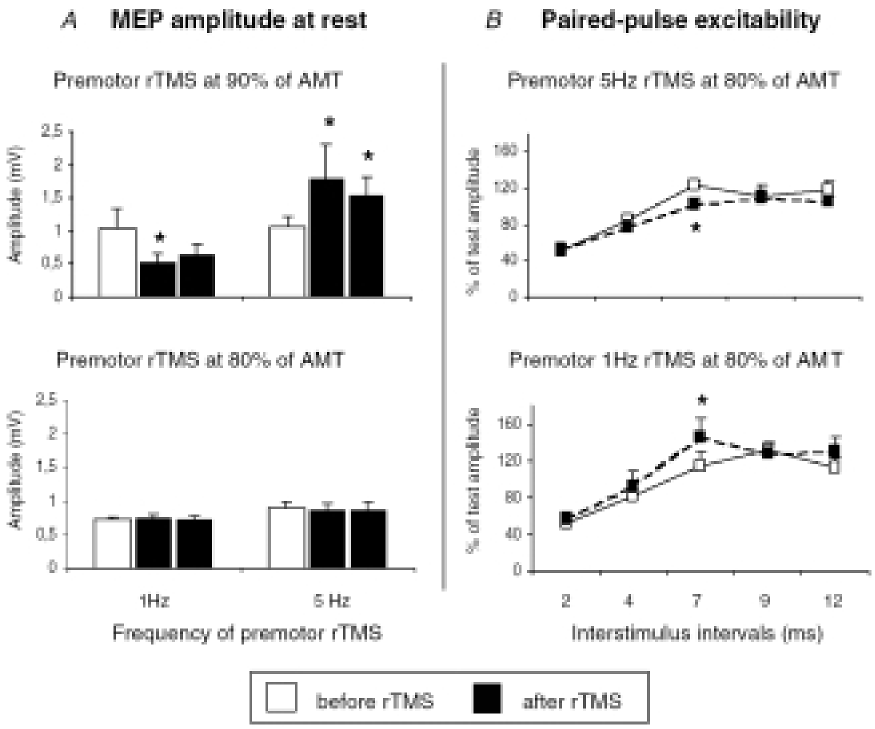

Fig. 1. Interactions between frequency and intensity of premotor rTMS on conditioning effects in the left M1hand. A: Conditioning effects on corticospinal excitability as indexed by the MEP amplitude at rest. The columns indicate mean MEP amplitudes before (white) and after premotor rTMS (black). B: Conditioning effects of premotor rTMS at 80% of AMT on paired-pulse excitability at an ISI of 7 ms. Error bars are standard error of the mean (S.E.M.). Asterisks denote a significant change relative to baseline. Used with permission from Rizzo et al. 2004 [68].

frequency band (coherence analysis). Local changes in frequency band amplitude can occur during cognitive tasks, providing information about local cortical activity; changes in coherence between distributed cortical areas provide a measure of interactions within a network of areas [58].

Imaging techniques such as positron emission tomography (PET) and the blood oxygen level dependent (BOLD) signal acquired in functional magnetic resonance imaging (fMRI) provide indirect measures of neuronal activity. These techniques rely on the coupling between increased synaptic activity and increased oxygen and glucose consumption (rCMR-glc, measured with [$^{18}$F] FDG-PET); and subsequent increases in cerebral blood flow (CBF) (measured with $H_2^{15}O$ PET and BOLD fMRI). Although a relationship between neuronal activity, metabolic rate and blood flow undoubtedly exists, the specific details are far from clear. For reviews on the subject see Raichle, Bonvento et al. and Logothetis and Wandell [9,44,65]. Neuroimaging measures appear to be convincingly coupled with synaptic activity and therefore enable the investigation of remote cortical and subcortical influences of TMS. Changes in local field potential are correlated with increased CBF, implying that local processing of inputs is the driving force for changes in cortical haemodynamics and hence the BOLD signal [39,40,46,47, 57]. By contrast, increases in neuronal firing rate may not be the best predictor for increases in either rCBF or BOLD [39,40,43].

Areas where activity is altered by TMS can be identified in a number of ways. Using PET or SPECT a simple contrast of pre and post rTMS sessions can be used to identify areas where synaptic activity has increased or decreased as a result of rTMS with the subject at rest. Alternatively, linear contrasts can be used to identify areas where activity was increased (or decreased) by rTMS and is returning to a baseline level of activity over the time course of the scanning session. It is also possible to use a factorial design to explore interactions between the effects of rTMS on resting synaptic activity and task-related activity by comparing changes in task-related activity (movement minus baseline) between pre and post rTMS sessions. When using fMRI to explore the enduring effects of rTMS it is only possible to use this third category of experiment design because of the confounding effects of different sessions on mean activity. The three design categories yield complementary information about the effects of rTMS on synaptic activity.

It is important to establish a 'proof of principle', demonstrating that it is possible to detect local effects of TMS at subthreshold intensities. Subthreshold TMS alters the responsiveness of the cortex to subsequent stimuli; therefore it should be possible to detect changes in synaptic activity using imaging techniques. The use of suprathreshold TMS in the motor system is more problematic because, by definition, suprathreshold stimuli will cause movement and therefore re-afferent feedback will contribute to any changes seen at the site of stimulation. Recording EEG activity over the whole cortex Ilmoniemi et al. [34] showed that single subthreshold TMS pulses elicited an immediate increase in local activity, spreading to adjacent ipsilateral motor and premotor areas within 3–10 ms, and to the homologous contralateral M1 within 20 ms. Using $H_2^{15}O$ PET Siebner et al. [80] found frequency dependent changes in regional cerebral blood flow (rCBF) restricted the site of stimulation (M1) during short trains of subthreshold stimulation (1–5 Hz rTMS). Takano et al. (2004) report increases in rCBF at the site of stimulation (M1) following short trains of subthreshold 5 Hz rTMS. The magnitude of the rCBF changes correlated with changes in paired-pulse excitability, suggesting that rCBF is sensitive to changes in excitability measured using direct techniques. Given that it is possible to measure local effects of TMS with functional imaging techniques, we can now examine distributed effects. These are discussed in two categories: immediate effects of single pulse and repetitive TMS measured during stimulation and enduring effects of rTMS measured after stimulation.

### 3.1. *Immediate effects of TMS measured with functional imaging*

Using ERP and spectral analysis techniques Paus et al. [61] demonstrated that single suprathreshold pulses of TMS led to distinctive local ERP waveforms (P30 and N45) and brief periods of increased activity in the beta range (15–30 Hz) at the site of stimulation. A series of technically challenging experiments [7,8] report increases in BOLD signal in the stimulated M1 measured in the MRI scanner during 1 Hz rTMS at suprathreshold intensities, and in the stimulated and contralateral M1 in response to single TMS pulses. Several studies have investigated BOLD responses during short trains of sub- and suprathreshold rTMS at a range of frequencies (3, 4, and 10 Hz) using three different field strengths (1.5 T, 2 T and 3 T). No changes in BOLD signal were observed at the site of stimulation with subthreshold 4 Hz rTMS at 1.5 T [5] or 3 Hz rTMS at 3 T [6]. In both studies local BOLD signal changes occurred only with suprathreshold stimuli. Distributed increases in BOLD signal were seen during both sub- and suprathreshold rTMS trains in the supplementary motor area (SMA) and premotor cortices, and decreases in BOLD signal were seen in the contralateral M1. Localised subcortical activations were observed in the putative motor thalamic nuclei and basal ganglia. Simultaneous recordings of electromyographic activity confirmed that these were not caused by covert peripheral muscle movements [6] see Fig. 2. At 2 T no BOLD signal changes were seen in M1 or premotor cortex with subthreshold stimulation to M1 (10 Hz rTMS) or suprathreshold stimulation to premotor cortex, whereas BOLD signal changes were detected in M1 during suprathreshold stimulation and voluntary finger movement. BOLD signal changes were seen in the SMA and premotor cortex with suprathreshold stimulation [3]. These functional neuroimaging studies demonstrate that short subthreshold stimulus trains are able to evoke distributed changes within cortical and subcortical motor structures.

A further examination of the effects of intensity (sub- to suprathreshold) with 1 Hz rTMS [82] reports increases in rCBF (measured with H215O PET) at site of stimulation (M1), contralateral cerebellum and in bilateral sub-cortical structures. The data presented in this paper suggest a non-linear relationship between intensity and rCBF at the site of stimulation: subthreshold stimulation appears not to evoke a significant increase in rCBF; whereas suprathreshold stimulation does.

### 3.2. *Enduring effects of rTMS measured with functional imaging*

Different frequencies of rTMS delivered to primary and premotor cortices can alter the excitability of distributed brain areas. Suprathreshold 1 Hz rTMS to left M1 lead to increased rCBF during stimulation that slowly decreased in magnitude after cessation of stimulation. Positive correlations with the rCBF changes at the site of stimulation were seen in the ipsilateral sensory and premotor areas and contralateral SMA while negative correlations were seen in contralateral M1 [26]. Subthreshold 5 Hz rTMS to M1 induced lasting increases in synaptic activity (measured using

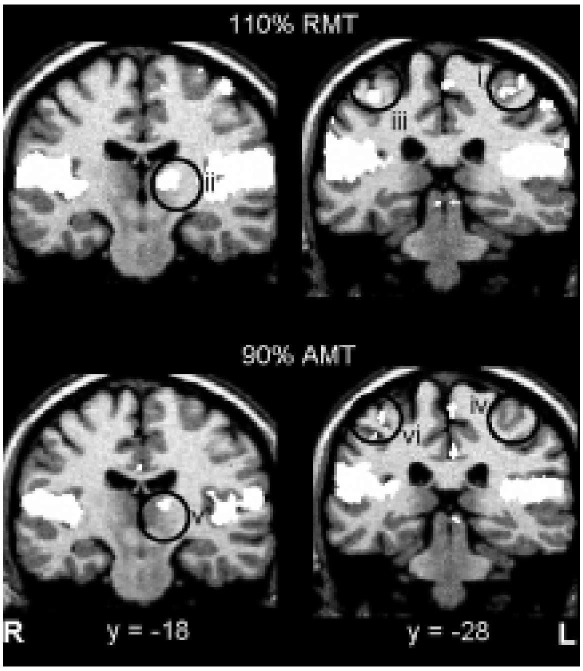

Fig. 2. Differences in the effects of sub- and suprathreshold rTMS on BOLD signal. BOLD signal increases during (top) suprathreshold rTMS (110% RMT), and (bottom) subthreshold rTMS (90% AMT). Apart from pronounced auditory activation, suprathreshold rTMS induced activations (red-yellow) in the left M1/S1 (i), and the left motor thalamus (ii). BOLD MRI signal decreases (iii) were observed in the right M1/S1. In the absence of peripheral muscle movements, subthreshold rTMS did not evoke significant changes at the site of stimulation (iv), while clear activity increases were observed in the left motor thalamic regions (v). A signal decrease was observed in the right M1/S1 (vi). $N = 11$, $P < 0.01$ (corrected). Results are projected onto two coronal sections of a standard reference brain (MNI coordinates indicated). Used with permission from Bestmann et al. 2004 [6].

[$^{18}$F] FDG-PET) at the site of stimulation and the SMA, with increased rCMR-glc also observed in contralateral M1 [79].

Comparing the effects of subthreshold rTMS 1 Hz to M1 and premotor cortex Chouinard et al. [17] correlated changes in MEP amplitude with changes in rCBF. Following 1 Hz rTMS to M1 positive correlations with decreased MEP amplitude were seen in the contralateral M1, ipsilateral cerebellum, cingulate motor area and sub-cortical structures. Following subthreshold 1 Hz rTMS to left premotor cortex widespread positive correlations with decreased MEP amplitude were seen bilaterally in the ventral premotor areas, cingulate motor areas, subcortical structures and a range of prefrontal and parietal regions. The authors inferred that areas showing parallel changes in rCBF with MEP amplitude are likely to be anatomically connected to site of stimulation (based on the macaque literature). It is also of note that 1 Hz rTMS to primary and premotor cortex had similar effects on MEP amplitudes, but that there was minimal spatial overlap between observed changes in rCBF.

Lee et al. [41] and Siebner et al. [77] examined the effects of subthreshold 1 Hz rTMS to M1 and PMd respectively, using the same experimental paradigm. In both experiments, changes in rCBF were detected that lasted for at least one hour after the end of stimulation. The different stimulation sites lead to profound differences in the direction and location of rCBF changes: M1 stimulation lead to bilateral increases in rCBF in primary and premotor areas and the cerebellum, whereas PMd stimulation lead to widespread bilateral decreases in rCBF in premotor, prefrontal, primary motor and subcortical areas.

In the first published work using fMRI to examine the enduring effects of rTMS Tegenthoff et al. [85] correlated the effects of 5 Hz rTMS (delivered to left primary sensory cortex prior to scanning) on tactile discrimination thresholds of the right index finger (measured in a separate experimental session), and cortical activity induced by electrical stimulation of the right index finger, suggesting that 5 Hz rTMS results in an expansion of the index-finger representation in the primary sensory cortex that may be related to the improvement in tactile discrimination.

Functional neuroimaging does not consistently detect changes in synaptic activity at the site of stimulation following subthreshold stimulation; even at intensities which are known, from electrophysiological data, to affect excitability. This may reflect the incomplete characterisation of the relationship between changes in neuronal activity, cortical excitability and metabolic measures of synaptic activity, and is worthy of further investigation. The majority of studies report changes in regions that are known to be connected with the site of stimulation (based on the animal literature) and which are known from other functional imaging studies to be involved in motor tasks. Even in the absence of motor behaviour, changes are seen throughout the motor network.

The results outlined in Sections 1 and 2 confirm that the two approaches to measuring changes in activity and excitability provide complementary information. Changes in excitability or synaptic efficacy determined using direct measures, i.e., changes in MEP amplitude do not have a straightforward relationship with measures of synaptic activity, measured with functional imaging. Moreover, neither method provides any in-

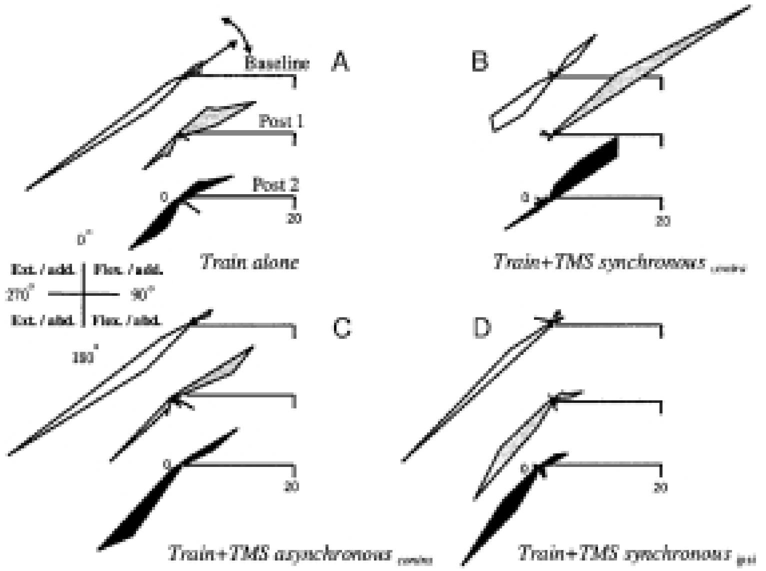

Fig. 3. Effects of Single TMS pulses to M1 (ipsilateral and contralateral to the moving hand) during training on direction of TMS-evoked movements. TMS-evoked movement directions from a single subject displayed as circular histograms at baseline and at two points after training. Mean training angle (arrow) and target training zone for all conditions are shown in A. In the Train alone (A) and Train + TMS asynchronous-contra (C) conditions, TMS-evoked movements at baseline were mainly in the extension/abduction (ext/abd) direction (inset). At post 1, there was a substantial increase in the proportion of TMS-evoked movements in the target training zone (flex/add). After 10–20 min (post 2), most TMS-evoked movements returned to the baseline direction. Note that Train + TMS synchronous-contra (B) elicited a more prominent and longer lasting change and that Train + TMS synchronous-ipsi (D) blocked the training effects. Used with permission from Butefisch et al. 2004 [10].

formation about the functional relevance of these alterations in synaptic activity and efficacy. For this it is necessary to use measures of motor performance.

## 4. Changes in motor behaviour

TMS can be used to disrupt or enhance motor performance in two modes: an acute disruptive mode (single-pulse or short trains of high-frequency rTMS) or a conditioning mode (prolonged trains of rTMS).

Acute disruptive effects of premotor TMS have been studied in a series of experiments using simple and choice reaction tasks [75]. An asymmetry in premotor contribution to task performance was demonstrated: TMS to left premotor cortex at short, but not long, cue-stimulus intervals increased reaction times for right and left handed responses in a choice reaction time task; whereas TMS to right premotor cortex only increased reaction times for left handed responses. During simple reaction time tasks, TMS did not affect reaction times. The effects of TMS on motor performance depend on the timing and location of TMS and the type of task being performed. The disruptive effect of TMS may also occur by affecting task related activity in a connected area. Meyer and Voss [50] showed that appropriately timed suprathreshold stimuli to the primary motor hand area can delay ballistic hand movements performed with the ipsilateral hand.

TMS delivered during motor behaviour may fail to show any effect of task performance. The area stimulated may not be uniquely involved in a particular aspect of the task being tested or other areas can compensate for the TMS induced disruption. Alternatively, the stimulated area may be crucial for task performance but stimulation parameters are inadequate to produce a substantial perturbation (e.g., intensity too weak, suboptimal coil orientation) or the timing of the stimulus is incorrect, i.e., the area is stimulated at a time when it is not participating in the task. These factors complicate the interpretation of null results.

Prolonged trains of rTMS can be used to alter motor behaviour by inducing lasting changes in the responsiveness of the stimulated cortex and connected areas. Lasting modulation of motor performance by rTMS conditioning also requires stimulation of an area involved in task performance. However, modulating the excitability of the primary motor cortex, known from imaging and primate experiments to be active across a huge range of motor tasks, has met with limited success in altering task performance. Despite the well documented effects of 1 Hz rTMS on the excitability of corticomotor projections [13,45,69,87,88], no impairment of manual motor control by 1 Hz rTMS has been convincingly demonstrated during simple motor tasks, e.g., paced fist clench [60], finger tapping [13,41,90], generation of freely selected movement sequences [41], maintenance of tonic contraction [84] peak force and acceleration during finger pinch [53]. 1 Hz rTMS to the premotor cortex has also failed to impair finger tapping and generation of freely selected movement sequences [77].

Recent work has revealed effects of rTMS on more demanding motor behaviour. Subthreshold 1 Hz rTMS to M1 decreased finger tapping rates when subjects were asked to tap as fast as possible with their right (dominant) hand and in tapping rates for both hands when tapping at subjects' fastest comfortable pace [35]. Subthreshold 1 Hz to left motor and premotor cortices increased reaction times in a 'masked prime' task [74]. In both studies, the authors infer that the tasks used were harder than those reported in previous work; leading to deficits in motor performance. This suggests that the motor system may be able to compensate, to some extent, for changes in cortical excitability during simple tasks, but not during more demanding behaviour. This concept of compensatory changes in response to alterations in motor excitability is discussed in Sections 4 and 5.

Suprathreshold 1 Hz rTMS impairs early consolidation of motor learning using a simple ballistic movement task [1,54]. An additional motor learning experiment showed that the same rTMS protocol did not disrupt learning of a dynamic force field [1]. These two studies serve to emphasise that the effects of rTMS conditioning on motor behaviour can be extremely task specific.

1 Hz rTMS appears to have opposite effects on the excitability of the stimulated and non-stimulated primary motor cortex which leads to differences in the effect on motor performance, specifically, an improvement in performance of a sequential key-pressing task with the hand ipsilateral to the stimulated hemisphere, and no change in performance with the hand contralateral to the site of stimulation [37]. The authors postulate that this may be due to a 'release' from the transcallosal inhibition imposed by the stimulated hemisphere. Differential effects of ipsilateral and contralateral TMS pulses can also be seen during motor learning [10]. Subjects were required to make repetitive thumb movements in the opposite direction to movements induced by TMS. Single pulses of subthreshold TMS were delivered to the primary motor cortex contralateral or ipsilateral to the moving hand during training. TMS pulses delivered contralateral to the moving hand, synchronously with the movements significantly enhanced the motor memory developed by the training period whereas TMS pulses delivered to the primary motor cortex ipsilateral to the moving hand lead to a failure to encode the motor memory seen with training alone (see Fig. 3). The authors postulate that synchronous TMS pulses delivered to contralateral M1 may enhance training by enhancing Hebbian plasticity; whereas TMS delivered to the ipsilateral M1 may enhance interhemispheric inhibition, thus decreasing the potential for training induced plasticity.

## 5. Combining functional measures of performance with measures of excitability and synaptic activity

In the motor system, it is possible to investigate changes in synaptic activity during task performance to assess how known changes in excitability affect movement related activity and connectivity. Chen et al. [16] used simple paced finger tapping and tonic contraction to examine the effects of subthreshold 0.9 Hz rTMS to premotor cortex on task-related EEG-power and EMG coherence. Decreases in power in the alpha and beta bands during the tasks were seen prior to rTMS; these were reduced for prolonged periods after stimulation. Increased task-related coherence was seen among cortical motor areas in upper alpha band frequencies; and decreased cortico-muscular coherence. Following rTMS, changes in spectral power at rest were restricted to the lower alpha-band. The authors infer that rTMS to the premotor cortex suppressed activation of motor areas during voluntary movement and suggest that transient reorganisation of movement-related activity in motor areas may occur.

Following subthreshold 1 Hz rTMS to primary motor cortex changes in EEG spectral power and coherence are seen: increases in EEG coherence between the motor and premotor areas ipsilateral to the site of stimulation, and increased coherence between the stimulated and contralateral primary motor areas in the alpha band during tonic contraction but not during rest [84]. Using the same protocol, short trains of subthreshold 5 Hz rTMS to M1 causes decreased intrahemispheric EEG coherence in the alpha band between motor and premotor cortices ipsilateral to the site of stimulation.

(a) (b)

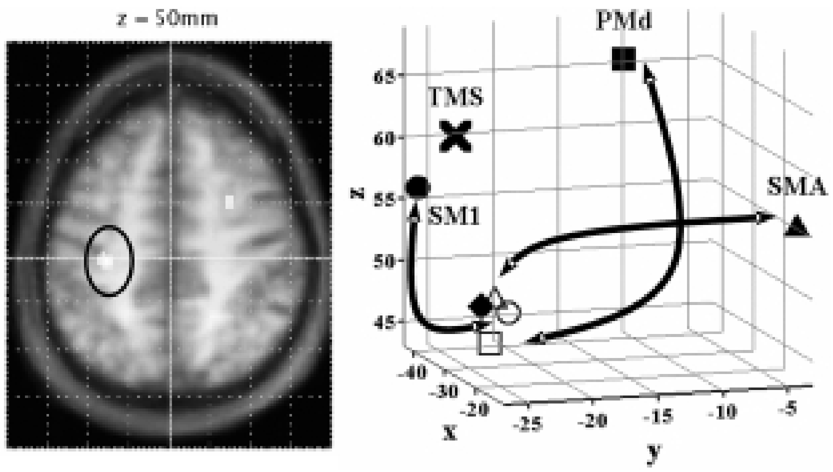

Fig. 4. Areas of the brain showing differential movement-related responses and coupling after rTMS. 4a) Increased movement related activity after 1 Hz rTMS. Results displayed on axial section of averaged anatomical MRI scans at $P < 0.001$ (uncorrected). 4b) Circle, square and triangle symbols indicating sites in primary motor cortex (open) that are more strongly coupled to activity in SM1, PMd and SMA (solid) respectively after rTMS. The solid diamond indicates the position of the SM1 site seen in 4a. 'X' marks the site of stimulation with 1 Hz rTMS. Adapted from Lee et al. [41]. Copyright 2003 by the Society for Neuroscience.

These changes were seen during tonic contraction, but not at rest [59]. These opposite effects of excitatory (5 Hz) and inhibitory (1 Hz) rTMS on alpha-band coherence agree with electrophysiological data demonstrating differing effects of 1 Hz and 5 Hz rTMS on local and distributed cortical excitability [30,55,68] because increases in alpha-band coherence are indicative of increased inhibitory activity, and decreases in alpha-band coherence are an indicator of reduced inhibitory activity (i.e. increased excitability).

Lee et al. [41] examined the effects of subthreshold 1 Hz rTMS delivered to Left M1 using PET. Following rTMS, task-dependent increases in rCBF (during movement of the right hand) were seen in the left primary motor cortex deep in the central sulcus (ipsilateral to the site of stimulation) and the dorsal premotor cortex in the contralateral hemisphere, with no change in motor performance. Additional analyses of effective connectivity suggest that after rTMS there is a re-modelling of the motor system, with increased movement-related connectivity from the SMA and premotor cortex to the sites in primary sensorimotor cortex that are unlikely to have been affected by rTMS (see Fig. 4).

Operational remapping of motor representations [16, 41], recruitment of additional motor areas (Fig. 4) [41] and task related changes in cortico-cortical [16,41, 59,84] and cortico-muscular [16] coherence may reflect compensatory changes in the motor system enabling maintenance of task performance during rTMS-induced changes in excitability. Mapping these patterns of reorganisation in the motor system may provide a useful method to study acute compensatory plasticity of the human brain and may help to understand how the brain reacts in response to more permanent lesions. However, in order to confirm that the changes in motor activity during movement after rTMS are functionally relevant (i.e. to distinguish compensatory changes from rTMS perturbations with no behavioural consequences) it is necessary to combine studies such as those described in this section with the approaches described in Section 3. This approach has already had some success with studies of stroke patients, where functional imaging has been used to identify cortical areas with increased activity during movement and TMS has then been used to stimulate these areas during movement to test the functional relevance of the increased activity. In this way it has been shown that disrupting activity in the contralesional premotor [36] and ipsilesional motor [91] and premotor [27] cortices impairs motor performance in stroke patients. Strens et al. [83] used a finger tapping task requiring precise force generation to show that stimulating either the primary motor cortex contralateral or ipsilateral to the hand performing the task with short trains of subthreshold 5 Hz rTMS had very limited effects on task performance; whereas stimulating both primary motor cortices simultaneously had a marked, prolonged, detrimental effect on finger tapping performance (see Fig. 5). The authors

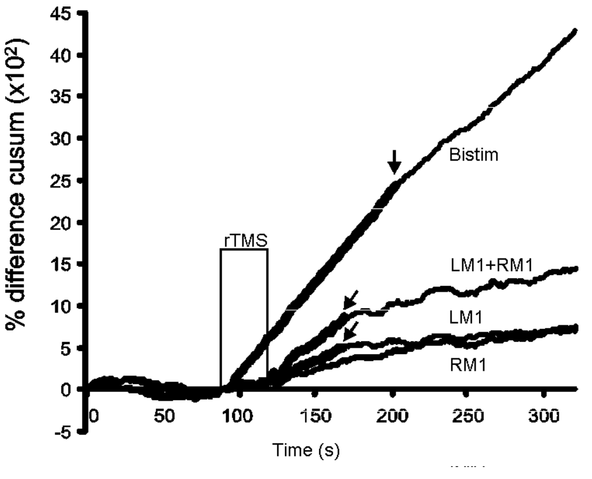

Fig. 5. Cumulative effects of simultaneous ipsilateral and contralateral rTMS on finger tapping. Average cumulative sum ($n = 7$) of the percentage difference in peak force compared to baseline (effects of occipital stimulation subtracted). The tapping force became inappropriately elevated after rTMS was delivered to LM1 from 8 s (95% confidence limits 1–21 s) to 51 s (43–63 s) after rTMS. RM1 stimulation did not elicit any significant change, and the algebraic sum of the effects of rTMS over the contralateral and ipsilateral motor cortices (LM1 + RM1) had the same onset and duration as the effect of contralateral stimulation alone. The effect of simultaneous bilateral stimulation of both motor cortices began during stimulation and continued until 86 s (69–102 s) after rTMS. Used with permission from Strens et al. 2004 [83].

suggest that when one primary motor cortex is acutely disrupted during motor performance, the other motor cortex provides functionally significant compensation i.e. that there is inherent redundancy in motor control systems. This suggests that the changes in movement-related activity following longer trains of rTMS reported in other studies may also represent functionally significant compensatory activity, although this remains to be formally tested.

## 6. Improving and impairing motor function with TMS

The work reviewed in Sections 3 and 4 shows that the effects of TMS on excitability and synaptic activity reviewed in Sections 1 and 2 are more likely not to change motor behaviour than to impair or improve task performance. The findings of combined studies of motor behaviour and functional neuroimaging reviewed in Section 4 reveal potential mechanisms by which the motor system may be able to compensate for excitability changes and maintain performance during simple tasks. The work of Strens et al. [83] suggests that simultaneous stimulation of multiple areas engaged in task performance, drawn from neuroimaging studies of movement-related changes, may provide a more effective method of impairing motor function with rTMS.

An important factor to consider when looking for improvements in motor performance after modulation of cortical excitability is 'ceiling' effects. If some studies of motor behaviour following rTMS fail to detect impaired performance because the tasks are too simple; it may be equally difficult to detect improvements in performance if subjects are performing 'perfectly' prior to an intervention. It is necessary to use tasks, or subjects, with scope for improvement.

The effects of TMS on motor behaviour may be more readily observed in groups of subjects with compromised cortical function. For example, patients with focal dystonia have abnormal cortical excitability [25,66] and show abnormal rCBF changes following rTMS [77]. In these subjects rTMS has been shown to normalise cortical excitability and improve motor function [76,81]. As outlined in Section 4, stroke patients appear to have increased susceptibility to the acute disruptive effects of TMS during motor tasks [27,36].

Changes in motor behaviour can themselves alter the excitability of the motor cortex [52]. In addition, it has also been shown in humans [92] and animals [67] that intensive motor training alters the efficacy with which subsequent conditioning paradigms modulate cortical excitability in the circuits engaged in motor learning. The work of Butefisch et al. [10] suggests that the reverse may also apply, i.e., modulation of a specific cortical circuit affects the rate and degree of change of motor behaviour.

Kobayashi et al. [37] highlight another potential method of improving motor function in healthy and abnormal brains. These researchers stimulated a motor area not directly involved in the task, reducing the impact of the stimulated area on the core areas involved in task performance. This suggests that more subtle, indirect approaches that aim to alter the balance of interactions among brain areas may be effective at improving function, and underlines the fact that researchers should be careful when selecting 'control regions' for TMS experiments in order to avoid selecting areas that may have an influence on the site of primary interest. Within the motor system the availability of direct measures of cortical excitability, such as interhemispheric inhibition, can guide such interventions.

One final method of increasing the impact of rTMS on motor behaviour would be to increase the impact of stimulation on cortical excitability. Pre-conditioning or priming the motor cortex with transcranial direct current stimulation enhances the duration and efficacy of 1 Hz rTMS on cortical excitability [78]. Priming the motor cortex with subthreshold 6 Hz rTMS significantly increases the duration and amount of cortical depression induced by suprathreshold 1 Hz rTMS. When two sessions of 1 Hz rTMS are delivered to the premotor cortex on consecutive days the effects on motor excitability have a longer duration [4]. This suggests that the distributed effects of premotor stimulation are not restricted to an immediate modulation of motor excitability, but creates a "motor memory" which primes the responsiveness of the cortex to a second session of rTMS. While none of these studies examined the effects of the various interventions on motor behaviour, it is possible that paradigms with profound and consistent electrophysiological effects may produce more consistent and readily detectable behavioural effects.

## 7. Conclusion

Single transcranial magnetic stimuli delivered to a discrete cortical area can alter the responsiveness of a remote site for a period of several hundred milliseconds. The direction of the change in responsiveness (facilitation or inhibition) depends on the timing and intensity of the conditioning and test stimuli. Changes in excitability on such short timescales are a valuable method of discerning the relationship between activity in distinct cortical areas (such as homologous primary motor cortices) in the healthy brain, in disorders of abnormal cortical excitability (e.g. dystonia) and after brain injury, such as stroke. They are unlikely to represent a clinically relevant method of modulating brain activity to improve function. Trains of repetitive transcranial magnetic stimuli have more enduring effects on the responsiveness of local and remote sites. The direction of change in responsiveness in distributed brain regions may differ from the changes seen at the site of stimulation; thus altering the relationship among neuronal activities in the affected areas.

The enduring changes in responsiveness induced by rTMS lead to changes in the pattern of distributed synaptic activity at rest, and during a range of motor tasks. For some types of motor behaviour it has been possible to impair or improve performance following rTMS, but for most simple tasks performance is unaltered. It is therefore possible that changes in distributed activity observed with functional neuroimaging during motor behaviour may represent compensatory activity, enabling maintenance of performance. Stimulation of additional cortical areas in order to disrupt compensatory activity appears to impair performance. Future attempts to modulate motor behaviour with rTMS should consider the widespread changes in synaptic activity, the possibility that this may represent functionally significant plasticity within the motor system and the potential benefits of indirect stimulation of areas involved in task performance or the use of stimulation protocols that enhance the efficacy of subsequent rTMS.

## Acknowledgements

This work was supported by the Wellcome Trust (LL, SB) and the Volkswagenstiftung (HRS).
